# Rising trends in obesity prevalence among Royal Thai Army personnel from 2017 to 2021

**DOI:** 10.1038/s41598-022-11913-2

**Published:** 2022-05-11

**Authors:** Boonsub Sakboonyarat, Jaturon Poovieng, Kanlaya Jongcherdchootrakul, Phutsapong Srisawat, Panadda Hatthachote, Mathirut Mungthin, Ram Rangsin

**Affiliations:** 1 Department of Military and Community Medicine, Phramongkutklao College of Medicine, Bangkok, 10400 Thailand; 2 Department of Medicine, Phramongkutkalo College of Medicine, Bangkok, 10400 Thailand; 3 Department of Physiology, Phramongkutklao College of Medicine, Bangkok, 10400 Thailand; 4 Department of Parasitology, Phramongkutklao College of Medicine, Bangkok, 10400 Thailand

**Keywords:** Risk factors, Epidemiology, Public health

## Abstract

Obesity is an essential health issue leading to noncommunicable diseases (NCDs) as well as atherosclerotic cardiovascular diseases. We aimed to determine the trends in obesity prevalence among Royal Thai Army (RTA) personnel and their associated factors using the health examination of RTA personnel database. A series of cross-sectional studies were conducted from 2017 to 2021. A self-report guide was created using a standardized case report form to obtain demographic characteristics and determine behavioral risk factors. Obesity was defined as BMI $\ge$ 25 kg/m^2^, and a total of 512,476 RTA personnel nationwide were included. Obesity prevalence rose from 42.1% (95% CI 41.7–42.4) in 2017 to 44.2% (95% CI 43.9–44.5) in 2021 (*p* for trend < 0.001). A significant surge was observed in obesity prevalence among young RTA personnel aged 18–24 years from 23.7% in 2017 to 28.4% in 2021 (*p* for trend < 0.001). Higher age individuals, male participants and RTA personnel residing in Bangkok tended to have a significantly higher risk for obesity. Further, regular exercise was a protective factor for obesity. Our data emphasized that obesity among the RTA personnel has been continuously rising over one half-decade, especially among young adults.

## Introduction

Obesity is one of the essential causes of noncommunicable diseases (NCDs) including type 2 diabetes (T2D), cancer, hypertension (HT) as well as atherosclerotic cardiovascular diseases (ASCVD)^1–5^. In addition, obesity was associated with an increase in the number of chronic diseases as a dose–response relationship^6^. According to the World Health Organization (WHO) report in 2016, globally, adults aged 18 years and older with body mass index (BMI) > 25 kg/m^2^ totaled approximately more than 1.9 billion; moreover, of these over 650 million had BMI > 30 kg/m^2^^7^. The 5th National Health Examination Survey (NHES V) in 2014 indicated that obesity prevalence (BMI ≥ 25 kg/m^2^) in a Thai population aged ≥ 15 years was 32.9% and 41.8% among males and females, respectively^8^. Additionally, a recent study among young Thai men provided crucial evidence of rising trends in obesity prevalence from 2009 to 2016^4^.

Yearly, Nationwide, approximately 80,000 to 120,000 Royal Thai Army (RTA) personnel individuals participate in the yearly health examination provided by the RTA Medical Department (RTAMED). In the present study, we investigated the trends in obesity prevalence among RTA personnel using the physical health examination of RTA personnel database from 2017 to 2021. Furthermore, we also determined the associations between obesity and age, geographic region, and behavioral factors.

## Methods

### Study design and subjects

A series of cross-sectional studies were conducted from 2017 to 2021. The dataset was retrieved from the annual health examination database of RTA personnel after obtaining permission from the RTAMED in Bangkok, Thailand. The RTAMED provides annual health examinations for RTA personnel through the Army Institute of Pathology (AIP), Armed Forces Research Institute of Medical Sciences (AFRIMS) and 37 RTA hospitals nationwide; then the records are reported to the RTAMED in Bangkok. Approximately 80,000 to 120,000 RTA personnel access the health examination, annually. The inclusion criteria for this study included active-duty RTA personnel aged 18 to 60 years nationwide. Participants without health examination records each year were excluded.

### Data collection

Annually, AIP, AFRIMS and 37 RTA hospitals provide health examinations for RTA personnel. The RTA personnel’s body weight and height were measured to calculate BMI. A self-report guide was created using a standardized case report form to obtain demographic characteristics and behavioral risk factors. The participant’s body weight was measured in kilograms, and the height in centimeters. BMI was calculated as body weight in kilograms divided by height in meters squared (kg/m^2^).

### Outcome variables

BMI was classified in six groups, including < 18.50 kg/m^2^, 18.5 to 22.99 kg/m^2^, 23.00–24.99 kg/m^2^, 25.00 to 29.99 kg/m^2^, 30.00–34.99 kg/m^2^ and $\ge$ 35.00 kg/m^2^. Obesity was defined as BMI $\ge$ 25 kg/m^9^. The age variable of study participants was categorized in eight groups presented in Table 1. The geographic region of residence consisted of Bangkok, north, northeast, central and south. The health scheme of the study participants included Civil Servant Medical Benefit, Social Security and Universal Coverage. Smoking status was categorized in three groups, including (1) never smoked, (2) ex-smoker defined as being smoke-free for 12 months and (3) current smoker. Alcohol consumption was divided in three groups including (1) never consumed, (2) ex-drinker defined as alcohol-free for 12 months and (3) current consumer. Regular exercise was defined as exercise 30 min/day and at least 3 days/week.

**Table 1:** Demographic characteristic of participants from 2017 to 2021(N = 512,476).

Year	2017	2018	2019	2020	2021
Characteristics	n (%)	n (%)	n (%)	n (%)	n (%)
Total	83,361	98,300	113,856	114,608	102,351
**Sex**					
Male	76,646 (91.9)	89,696 (91.2)	104,329 (91.6)	103,277 (90.1)	93,832 (91.7)
Female	6715 (8.1)	8604 (8.8)	9527 (8.4)	11,331 (9.9)	8519 (8.3)
**Age (years)**					
18–24	7128 (8.6)	9481 (9.6)	11,550 (10.1)	10,856 (9.5)	9257 (9.0)
25–29	15,998 (19.2)	19,713 (20.1)	23,029 (20.2)	23,264 (20.3)	21,071 (20.6)
30–34	13,382 (16.1)	16,783 (17.1)	19,789 (17.4)	20,077 (17.5)	19,077 (18.6)
35–39	8525 (10.2)	10,430 (10.6)	12,561 (11.0)	13,634 (11.9)	13,877 (13.6)
40–44	8581 (10.3)	10,315 (10.5)	11,572 (10.2)	10,839 (9.5)	9544 (9.3)
45–49	7318 (8.8)	8188 (8.3)	9128 (8.0)	9808 (8.6)	9088 (8.9)
50–54	11,512 (13.8)	11,521 (11.7)	12,160 (10.7)	10,843 (9.5)	8615 (8.4)
≥ 55	10,917 (13.1)	11,869 (12.1)	14,067 (12.4)	15,287 (13.3)	11,822 (11.6)
Mean ± SD	39.1 ± 11.5	38.2 ± 11.3	38.0 ± 11.4	38.1 ± 11.5	37.6 ± 11.1
**Regions**					
Bangkok	11,005 (13.2)	15,193 (15.5)	17,386 (15.3)	17,956 (15.7)	8754 (8.6)
Central	27,842 (33.4)	36,079 (36.7)	37,697 (33.1)	41,803 (36.5)	40,389 (39.5)
Northeast	15,085 (18.1)	16,842 (17.1)	19,710 (17.3)	21,795 (19.0)	17,633 (17.2)
North	19,516 (23.4)	15,737 (16.0)	24,009 (21.1)	18,633 (16.3)	22,878 (22.4)
South	9913 (11.9)	14,449 (14.7)	15,054 (13.2)	14,421 (12.6)	12,697 (12.4)
**Health scheme**					
Civil servant medical benefit	81,777 (98.1)	96,252 (97.9)	111,540 (98.0)	111,316 (97.1)	100,370 (98.1)
Social security	1019 (1.2)	1207 (1.2)	1429 (1.3)	2449 (2.1)	1673 (1.6)
Universal coverage	565 (0.7)	841 (0.9)	887 (0.8)	843 (0.7)	308 (0.3)
**Smoking status**					
Never	41,796 (52.5)	50,256 (52.2)	51,811 (46.9)	48,563 (44.9)	49,346 (48.5)
Ex-smoker	15,112 (19.0)	15,986 (16.6)	23,533 (21.3)	23,206 (21.4)	18,480 (18.2)
Current smoker	22,646 (28.5)	30,036 (31.2)	35,021 (31.7)	36,477 (33.7)	33,918 (33.3)
**Alcohol drinking**					
Never	18,196 (23.0)	22,003 (23.0)	22,775 (20.1)	18,698 (17.2)	23,521 (23.1)
Ex-drinker	7244 (9.2)	10,371 (10.8)	13,720 (12.1)	12,549 (11.6)	11,695 (11.5)
Current consumer	53,504 (67.8)	63,259 (66.1)	76,621 (67.7)	77,347 (71.2)	66,512 (65.4)
**Regular exercise**					
Yes	30,714 (39.2)	37,427 (39.9)	37,553 (34.0)	46,008 (41.9)	48,092 (47.3)
No	47,695 (60.8)	56,363 (60.1)	72,794 (66.0)	63,699 (58.1)	53,500 (52.7)
**Body mass index (kg/m**^2^**)**					
18.50–22.99	26,191 (31.4)	30,724 (31.3)	35,075 (30.8)	35,500 (31.0)	30,691 (30.0)
< 18.50	1841 (2.2)	2034 (2.1)	2305 (2.0)	2376 (2.1)	2050 (2.0)
23.00–24.99	20,270 (24.3)	23,807 (24.2)	27,461 (24.1)	27,169 (23.7)	24,413 (23.9)
25.00–29.99	28,009 (33.6)	32,932 (33.5)	38,298 (33.6)	38,285 (33.4)	34,592 (33.8)
≥ 30.00–34.99	5925 (7.1)	7397 (7.5)	8914 (7.8)	9379 (8.2)	8760 (8.6)
≥ 35.00	1125 (1.3)	1406 (1.4)	1803 (1.6)	1899 (1.7)	1845 (1.8)
Mean ± SD	24.7 ± 3.7	24.8 ± 3.8	24.9 ± 3.8	24.9 ± 3.9	25.0 ± 3.9

*SD* standard deviation.

### Statistical analysis

Statistical analyses were performed using StataCorp. 2021. *Stata Statistical Software: Release 17*. College Station, TX: StataCorp LLC. Baseline characteristics were calculated using descriptive statistics. Due to the nature of an observational study, some data of some variables were missing, including smoking status (3.2%), alcohol consumption (2.8%) and exercise (3.6%). Although we were aware of missing data, the study comprised a large sample size; thus, the existing data would be included in the analysis. Categorical variables including sex, age groups, regions, health scheme, smoking status, history of alcohol consumption, a history of exercise and BMI categories were presented as percentage. Continuous variables including age and BMI were presented as mean, standard deviation (SD). The outcome of the study was obesity prevalence among RTA personnel presented as a percentage and 95% confidence interval (CI). *P* for trend was calculated using *chi*-square statistics for trends. To determine the association between covariates and obesity, binary logistic regression analysis was performed, and the magnitude of association was presented as crude odds ratio (ORs) with 95% CI. A multiple logistic regression model was used to determine independent factors associated with obesity among RTA personnel. The variables in the final model included year, sex, age, regions, health scheme, smoking status, alcohol consumption and exercise. The magnitude of association was presented as adjusted odds ratio (AORs) with 95% CI. A *p*-value less than 0.05 was considered statistically significant.

### Ethics consideration

The study was reviewed and approved by the Institutional Review Board, RTA Medical Department in compliance with international guidelines such as the Declaration of Helsinki, the Belmont Report, CIOMS Guidelines and the International Conference on Harmonization of Technical Requirements for Registration of Pharmaceuticals for Human Use—Good Clinical Practice (ICH-GCP) (approval number S067h/64). Due to using secondary data, a waiver of documentation of informed consent was used, and the waiver for informed consent was granted by the Institutional Review Board, RTA Medical Department.

## Results

### Demographic characteristics

A total of 512,476 RTA personnel were included in the study from 2017 to 2021. Demographic data of participants are presented in Table 1. The average age of participants was 38.2 ± 11.4 years. In all, 467,780 participants (91.3%) were males. One third of all participants resided in the central region. Almost all participants were under the Civil Servant Medical Benefit scheme, approximately 98%. The prevalence of current smokers was 28.5% in 2017 while it reached 33.3% in 2021. In terms of alcohol consumption, approximately two thirds of participants were current drinkers over 5 years. Participants with a history of regular exercise were 39.2% in 2017, and rose to 47.3% in 2021. The average BMI of participants was 24.7 ± 3.7 kg/m^2^ in 2017, 24.8 ± 3.8 kg/m^2^ in 2018, 24.9 ± 3.8 kg/m^2^ in 2019, 24.9 ± 3.9 kg/m^2^ in 2020 and 25.0 ± 3.9 kg/m^2^ in 2021.

### Trends in obesity prevalence among the RTA personnel from 2017 to 2021

**Figure 1:**
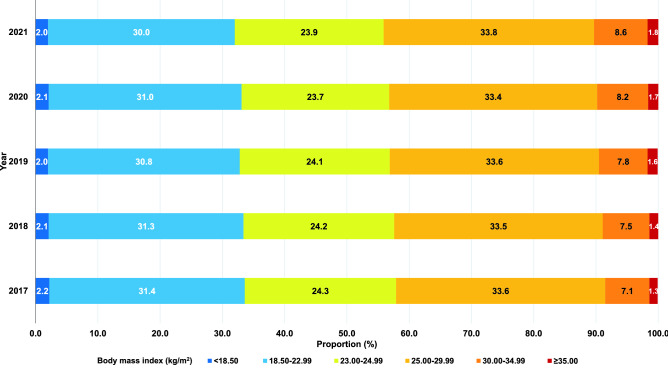
Proportion of BMI groups among RTA personnel from 2017 to 2021.

The proportion of the RTA personnel with BMI 25.00 to 29.99 kg/m^2^ rose from 33.6% in 2017 to 33.8% in 2021 while the proportion of the RTA personnel with BMI 30.00 to 34.99 kg/m^2^ significantly increased from 7.1 in 2017 to 8.6% in 2021. Furthermore, the prevalence of RTA personnel having BMI $\ge$ 35 kg/m^2^ was continuously incremental from 1.3% in 2017 to 1.8% in 2021. Figure 1 demonstrates the proportion of BMI groups among RTA personnel from 2017 to 2021.

**Figure 2:**
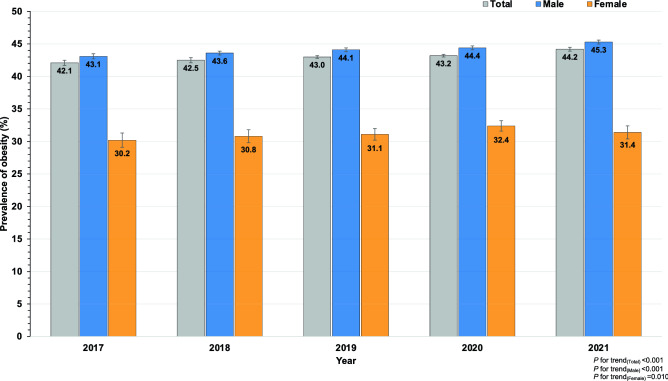
Prevalence of obesity (BMI $\ge$ 25 kg/m^2^) and 95% CI among RTA personnel by sex from 2017 to 2021.

From 2017 to 2021, the overall obesity prevalence among RTA personnel increased significantly by the year of the study. Table 2 illustrates the trends in obesity prevalence among RTA personnel by sex, age groups and regions over 5 years. Obesity prevalence rose from 42.1% (95% CI 41.7–42.4) in 2017 to 42.5% (95% CI 42.1–42.8) in 2018, 43.0% (95% CI 42.8–43.3) in 2019, 43.2% (95% CI 43.0–43.5) in 2020 and 44.2% (95% CI 43.9–44.5) in 2021 (*p* for trend < 0.001). Figure 2 presents obesity prevalence among RTA personnel by sex from 2017 to 2021. Obesity prevalence among male participants was higher than that among females over five years. Among males, obesity prevalence significantly increased from 43.1% in 2017 to 45.3% in 2021 (*p* for trend < 0.001) while, among females, it rose from 30.2% in 2017 to 31.4% in 2021 (*p* for trend = 0.010). In addition, obesity prevalence among ages 18 to 49 years significantly rose from 2017 to 2021 (*p* for trend < 0.05); moreover, a dramatic increase was noted in obesity prevalence among young RTA personnel aged 18 to 24 years by 4.7% over one half-decade (*p* for trend < 0.001). In terms of geographic region, obesity prevalence among RTA personnel residing in all regions, except in the south, significantly increased over five years (*p* for trend < 0.05).

**Table 2:** Trends in obesity prevalence (BMI ≥ 25 kg/m^2^) among RTA personnel from 2017 to 2021.

Characteristics	2017 (N = 83,361)		2018 (N = 98,300)		2019 (N = 113,856)		2020 (N = 114,608)		2021 (N = 102,351)		*p*-for trend
	n	% (95% CI)	n	% (95% CI)	n	% (95% CI)	n	% (95% CI)	n	% (95% CI)	
Overall	35,059	42.1 (41.7–42.4)	41,735	42.5 (42.1–42.8)	49,015	43.0 (42.8–43.3)	49,563	43.2 (43.0–43.5)	45,197	44.2 (43.9–44.5)	< 0.001
**Sex**											
Male	33,033	43.1 (42.7–43.4)	39,085	43.6 (43.3–43.9)	46,052	44.1 (43.8–44.4)	45,890	44.4 (44.1–44.7)	42,523	45.3 (45.0–45.6)	< 0.001
Female	2026	30.2 (29.1–31.3)	2650	30.8 (29.8–31.8)	2963	31.1 (30.2–32.0)	3673	32.4 (31.6–33.3)	2674	31.4 (30.4–32.4)	0.010
**Age (years)**											
18–24	1691	23.7 (22.7–24.7)	2435	25.7 (24.8–26.6)	3085	26.7 (25.9–27.5)	2938	27.1 (26.2–27.9)	2625	28.4 (27.4–29.3)	< 0.001
25–29	5718	35.7 (35.0–36.5)	7201	36.5 (35.9–37.2)	8564	37.2 (36.6–37.8)	8619	37.0 (36.4–37.7)	8138	38.6 (38.0–39.3)	< 0.001
30–34	5605	41.9 (41.1–42.7)	7153	42.6 (41.9–43.4)	8798	44.5 (43.8–45.2)	9026	45.0 (44.3–45.6)	8851	46.4 (45.7–47.1)	< 0.001
35–39	3943	46.3 (45.2–47.3)	4817	46.2 (45.2–47.1)	5848	46.6 (45.7–47.4)	6387	46.8 (46.0–47.7)	6577	47.4 (46.6–48.2)	0.039
40–44	4169	48.6 (47.5–49.6)	5113	49.6 (48.6–50.5)	5785	50.0 (49.1–50.9)	5418	50.0 (49.0–50.9)	4827	50.6 (49.6–51.6)	0.009
45–49	3492	47.7 (46.6–48.9)	3914	47.8 (46.7–48.9)	4481	49.1 (48.1–50.1)	4837	49.3 (48.3–50.3)	4493	49.4 (48.4–50.5)	0.004
50–54	5457	47.4 (46.5–48.3)	5501	47.7 (46.8–48.7)	5800	47.7 (46.8–48.6)	5177	47.7 (46.8–48.7)	4193	48.7 (47.6–49.7)	0.129
≥ 55	4984	45.7 (44.7–46.6)	5601	47.2 (46.3–48.1)	6654	47.3 (46.5–48.1)	7161	46.8 (46.1–47.6)	5493	46.5 (45.6–47.4)	0.431
**Regions**											
Bangkok	4677	42.5 (41.6–43.4)	6573	43.3 (42.5–44.1)	7553	43.4 (42.7–44.2)	7979	44.4 (43.7–45.2)	3996	45.6 (44.6–46.7)	< 0.001
Central	11,998	43.1 (42.5–43.7)	15,763	43.7 (43.2–44.2)	16,459	43.7 (43.2–44.2)	18,313	43.8 (43.3–44.3)	18,115	44.9 (44.4–45.3)	< 0.001
Northeast	6267	41.5 (40.8–42.3)	7042	41.8 (41.1–42.6)	8477	43.0 (42.3–43.7)	9424	43.2 (42.6–43.9)	8070	45.8 (45.0–46.5)	< 0.001
North	8157	41.8 (41.1–42.5)	6741	42.8 (42.1–43.6)	10,509	43.8 (43.1–44.4)	8081	43.4 (42.7–44.1)	9875	43.2 (42.5–43.8)	0.004
South	3960	39.9 (39.0–40.9)	5616	38.9 (38.1–39.7)	6017	40.0 (39.2–40.8)	5766	40.0 (39.2–40.8)	5141	40.5 (39.6–41.3)	0.070

### Associated factors of obesity among RTA personnel

Univariable and multiple logistic regression analyses were performed to determine the associated factors of obesity among RTA personnel (Table 3). The final model was adjusted for survey year, sex, age, geographic region, health scheme, smoking status, alcohol consumption and history of regular exercise. After adjusting for potential confounders, the results indicated a significant increase in annual obesity prevalence. Male participants had an increased risk of obesity compared with females (AOR 1.96 95% CI 1.89–2.00). Higher-aged participants tended to present obesity when compared with younger participants. RTA personnel in the central, northeast, northern and southern regions had a lower risk for obesity compared with those residing in Bangkok (AOR 0.97, 0.94, 0.91, and 0.81, respectively). Regarding behavioral factors, ex-smokers were more likely to be obese compared with participants who never smoked (AOR 1.14; 95% CI 1.12–1.16) while no association was observed between alcohol consumption and obesity. Moreover, RTA personnel who regularly exercised were at a significantly lower risk of obesity (AOR 0.82; 95% CI 0.81–0.83) than those having no history of regular exercise.

**Table 3:** Univariable and multivariable analysis of the association between demographic and behavioral factors and obesity prevalence among RTA personnel (2017–2021).

Variables	Unadjusted OR (95% CI)	*p*-value	Adjusted OR (95% CI)	*p*-value
**Years**				
2017	1		1	
2018	1.02 (1.00–1.04)	0.086	1.05 (1.03–1.07)	< 0.001
2019	1.04 (1.02–1.06)	< 0.001	1.08 (1.06–1.10)	< 0.001
2020	1.05 (1.03–1.07)	< 0.001	1.08 (1.06–1.10)	< 0.001
2021	1.09 (1.07–1.11)	< 0.001	1.10 (1.08–1.12)	< 0.001
**Sex**				
Male	1		1	
Female	0.58 (0.56–0.59)	< 0.001	0.51 (0.50–0.53)	< 0.001
**Age (years)**				
18–24	1		1	
25–29	1.64 (1.60–1.68)	< 0.001	1.64 (1.60–1.68)	< 0.001
30–34	2.21 (2.15–2.26)	< 0.001	2.22 (2.16–2.28)	< 0.001
35–39	2.44 (2.37–2.50)	< 0.001	2.46 (2.40–2.53)	< 0.001
40–44	2.75 (2.68–2.83)	< 0.001	2.78 (2.71–2.86)	< 0.001
45–49	2.64 (2.57–2.72)	< 0.001	2.65 (2.58–2.73)	< 0.001
50–54	2.55 (2.48–2.61)	< 0.001	2.55 (2.48–2.62)	< 0.001
≥ 55	2.44 (2.38–2.50)	< 0.001	2.44 (2.38–2.51)	< 0.001
**Regions**				
Bangkok	1		1	
Central	1.00 (0.99–1.02)	0.679	0.97 (0.95–0.99)	0.002
Northeast	0.97 (0.96–0.99)	0.009	0.94 (0.92–0.96)	< 0.001
North	0.97 (0.95–0.99)	0.002	0.91 (0.89–0.93)	< 0.001
South	0.85 (0.83–0.87)	< 0.001	0.81 (0.79–0.83)	< 0.001
**Health scheme**				
Civil servant medical benefit	1		1	
Social security	0.75 (0.71–0.78)	< 0.001	1.15 (1.09–1.21)	< 0.001
Universal coverage	0.74 (0.69–0.80)	< 0.001	0.94 (0.87–1.01)	0.092
**Smoking status**				
Never	1		1	
Ex-smoker	1.22 (1.20–1.24)	< 0.001	1.14 (1.12–1.16)	< 0.001
Current smoker	1.04 (1.02–1.05)	< 0.001	1.00 (0.99–1.01)	0.844
**Alcohol drinking**				
Never	1		1	
Ex-drinker	1.11 (1.08–1.13)	< 0.001	0.99 (0.97–1.01)	0.246
Current consumer	1.09 (1.07–1.10)	< 0.001	1.00 (0.98–1.01)	0.783
**Regular exercise**				
No	1		1	
Yes	0.83 (0.83–0.84)	< 0.001	0.82 (0.81–0.83)	< 0.001

*OR* odds ratio, *CI* confidence interval.

## Discussion

To our knowledge, this study represented the largest epidemiologic study of obesity prevalence and its associated factors among RTA personnel in Thailand over one half decade. These data provide crucial evidence of rising trends in obesity prevalence among RTA personnel from 2017 to 2021. Factors associated with obesity included being male, higher age, geographic region, smoking status and no regular exercise.

The average BMI among RTA personnel ranged from 24.7 in 2017 to 25.0 kg/m^2^ in 2021 increasing at an average of 0.6 kg/m^2^ per decade. This finding was comparable with the trends in adult BMI in a pooled analysis of 1698 population-based measurement studies from 200 countries^10^. Compared with the related study among Iranian military personnel, obesity prevalence in the current study was relatively high^11^. The present study reported the overall obesity prevalence among RTA personnel rose by 2.1% over five years which was compatible with the civilian trends in Thailand showing an increasing obesity prevalence from 37.5 in 2014^8^ to 42.2% in 2019^12^. Similarly, a related study among active-duty military personnel in the US illustrated that combined overweight and obesity (BMI $\ge$ 25 kg/m^2^) continuously increased from 50.6% in 1995 to 60.8% in 2008^13^.

Several studies have indicated that obesity prevalence among females was relatively high when compared with that among males^6,8,10,12^. In this study, male participants tended to present a higher risk for obesity approximately twice that of females corresponding to the obesity situation among military personnel in the US in that overweight and obesity prevalence among males was significantly higher^13^.

The present study emphasized that a significant rising obesity prevalence among RTA personnel aged 18 to 49 years over 5 years, especially among young RTA personnel aged 18 to 24 years, surged from 23.7 to 28.4% over one half decade. However, among older age groups, a deceleration was observed of rising obesity prevalence during the study period. Correspondingly, the NHES V and VI surveys showed rising trends in obesity prevalence in a Thai population aged 15 to 29 years approximately 7% over 5 years while remaining relatively lower among older Thai adults^8,12^. In addition, one study in the US demonstrated active-duty military personnel aged less than 25 years having BMI ≥ 25 kg/m^2^ rose by approximately 8.6% from 1995 to 2008 while it remained slowly incremental among those with higher age^13^. This could be due to the transformation of Thai society to being more industrial; thus, fast food and high-energy food is more available^4,14^. The dietary behavior and lifestyle of young adults may facilitate increasing BMI in this age group.

The current study presented that higher age participants tended to have obesity as a dose–response relationship, peaking at 40 to 44 years and dropping at age $\ge$ 50 years. This pattern was consistent with related studies in the US and China illustrating that obesity prevalence was greater among middle-aged individuals and declined among older people^13,15^. This phenomenon may be explained by reduced lean muscle mass associated with higher age^16^.

We found that the RTA personnel residing in Bangkok and the central region were inclined to exhibit obesity when compared with those in other regions. This occurrence was similar to a recent report that the BMI level of people residing in Bangkok and the central region of Thailand was relatively high^17^. In this area, several agriculture businesses facilitate more than sufficient dietary products combined with inappropriate patient dietary behaviors that might have encouraged increasing weight^17^. In addition, the NHES VI survey in 2019 reported the proportion of Thai populations aged ≥ 15 years in Bangkok and the central region consuming fast food was relatively high when compared with those in other regions. Moreover, Thais in Bangkok and the central region consumed less dietary fiber including fruits and vegetables among both males and females^12^.

Our study reported that ex-smokers were inclined to exhibit obesity. Related evidence indicated that weight gain usually occurred after smoking cessation leading to declined metabolic rate, increased caloric intake and changes in food preferences^18^. Furthermore, one related study in the US reported that weight gain among smokers who quit was more than that gained among current smokers, approximately 4.4 kg among males and 5.0 kg among females over a 10-year period^19^. Conversely, a recent study reported that smoking status was unassociated with obesity^4^. However, the finding was outweighed by the advantages of smoking cessation, which are much greater than the superfluous risk granted by the weight gain.

Finally, we found that regular exercise was a protective factor for obesity among RTA personnel similar to a related study among young Thai men^4^. Therefore, our study suggested that regular exercise, such as increased physical activity, running and weight training, should be encourage among RTA personnel to decrease BMI and alleviate the progression of NCDs including ASCVD^20,21^. However, the intensity of exercise should be modified appropriately for personal characteristics, especially among individuals with heart disease and elderly people^22,23^. Our findings emphasized that modifiable risk factors for obesity such as no regular exercise should be improved among RTA personnel. Among young RTA personnel ages 18 to 24 years, obesity prevalence speedily rose over five years; therefore, health interventions including raising awareness regarding reducing their weight and associated further complications such as T2D and HT, should be facilitated in these age groups. Further, modifying dietary behaviors and regular self-weighing can also play an essential role to control BMI level^24–26^.

Several limitations were encountered in this study. First, the study employed a serial cross-sectional design; therefore, the results could demonstrate only the association between factors and outcomes. Second, the present study was conducted among RTA personnel comprising a greater proportion of male participants accounting for 91.3%; however, the results reported the real situation in this study population. According to the observational study using the health examination database, behavioral risk variables were collected very broadly, e.g., total number of alcohol drinks, total number of cigarettes and smoking frequency and intensity of exercise were not recorded. However, the associations between factors and outcomes were able to be presented. Our study also exhibited significant strengths, including a representative, large sample of RTA personnel. Thus, our findings provided valuable insights regarding the prevalence, demographics and behavioral risk factors of obesity in Thailand. Furthermore, these data may contribute to strategies for the primary prevention of metabolic syndrome, T2D, HT and ASCVD in this population.

## Conclusion

Our data emphasized that obesity among RTA personnel has been continuously rising from 2017 to 2021, especially among young adults. RTA personnel residing in Bangkok and the central region tended to present a greater obesity prevalence. Regular exercise should be encouraged to alleviate the process of obesity, and dietary behaviors should be modified concurrently.

## Data Availability

Data cannot be shared publicly because the data set contains identifying information; additionally, the data belong to the Royal Thai Army Medical Department. Thus, ethics restrictions exist concerning the data set. Data are available from the Royal Thai Army Medical Department, Bangkok, Thailand for researchers meeting the criteria to access confidential data.

## Abbreviations

NCDs: Noncommunicable diseases
T2D: Type 2 diabetes
HT: Hypertension
ASCVD: Atherosclerotic cardiovascular diseases
BMI: Body mass index
RTA: The Royal Thai Army
RTAMED: The Royal Thai Army Medical Department
AIP: Army Institute of Pathology
AFRIMS: Armed Forces Research Institute of Medical Sciences
NHES: National Health Examination Survey
AOR: Adjusted odds ratio
CI: Confidence interval
SD: Standard deviation
